# Association of interleukin‐27 gene polymorphisms with susceptibility to HIV infection and disease progression

**DOI:** 10.1111/jcmm.14067

**Published:** 2019-01-10

**Authors:** Xiao‐Xia Pang, Shun‐Da Luo, Ting Zhang, Feng Shi, Chun‐Fang Wang, Xing‐Hong Chen, Yu‐Xia Wei, Li Qin, Jing‐Xi Wei, Xiao‐Qiong Luo, Jun‐Li Wang

**Affiliations:** ^1^ Youjiang Medical University for Nationalities Baise China; ^2^ Reproductive Medicine Center The Affiliated Hospital of Youjiang Medical University for Nationalities Baise China; ^3^ Department of Laboratory Medicine The Fourth People's Hospital of Nanning Nanning China; ^4^ Department of Laboratory Medicine The Affiliated Hospital of Youjiang Medical University for Nationalities Baise China

**Keywords:** disease progression, HIV infection, IL‐27, polymorphism

## Abstract

Interleukin‐27 (IL‐27) gene polymorphisms are linked to infectious disease susceptibility and IL‐27 plasma level is associated with HIV infection. Therefore, we aimed to investigate the association between IL‐27 polymorphisms and susceptibility to HIV infection and disease progression. A total of 300 patients with HIV infection (48 long‐term nonprogressors and 252 typical progressors) and 300 healthy controls were genotyped for three IL‐27 polymorphisms, rs17855750, rs181206, rs40837 which were performed by using multiple single nucleotide primer extension technique. Significant association was found between IL‐27 rs40837 polymorphisms with susceptibility to HIV infection (AG vs AA: adjusted OR = 1.60, 95% CI, 1.11‐2.30, *P *=* *0.012; AG+GG vs AA: adjusted OR = 1.44, 95% CI, 1.02‐2.03, *P = *0.038) and disease progression (LTNP: AG vs AA: adjusted OR = 2.33, 95% CI, 1.13‐4.80, *P = *0.021; TP: AG vs AA: adjusted OR = 1.50, 95% CI, 1.04‐2.24, *P = *0.030). Serum IL‐27 levels were significantly lower in cases compared to controls (*P *<* *0.001). There were lower serum IL‐27 levels in TPs than in LTNPs (*P *<* *0.001). We further found that LTNPs with rs40837 AG or GG genotype had lower serum IL‐27 levels than with AA genotype (*P *<* *0.05). The CD4^+^T counts in cases were significantly lower than controls (*P *<* *0.001). In contrast, individuals with rs40837 AG genotype had lower CD4^+^T counts than with AA genotype in cases (*P *<* *0.05). In addition, CD4^+^T counts in TPs were significantly lower than LTNPs (*P *<* *0.001). IL‐27 rs40837 polymorphism might influence the susceptibility to HIV infection and disease progression probably by regulating the level of serum IL‐27 or the quantity of CD4^+^T.

## 1 INTRODUCTION

Acquired immunodeficiency syndrome (AIDS), caused by human immunodeficiency virus (HIV) infection and characterized by reduction in CD4^+^T cells and subsequent immune function defects, is a worldwide infectious disease which seriously affect patients’ physical and mental health because of its incurable property. The disease progression of HIV infection includes long‐term nonprogression (LTNP) and typical progression (TP) in clinic.^1^ On average, most long‐term nonprogressors will progress to typical progression within 10 years without antiretroviral therapy, while a small number remain no progression maintaining CD4^+^T counts above (over) 350 cells/μl for decades.^2^, ^3^ Previous studies suggested that genetic variations in genes that control immune responses may play an essential role in the HIV infection and disease progression.^4^, ^5^ Therefore, the study of the association between various gene polymorphisms and HIV infection and disease progression has become a hot topic at present.

Interleukin‐27 (IL‐27) composed of Epstein‐Barr virus‐induced gene (BBI3) and p28 protein, a member of IL‐12 family,^6^ is a heterodimeric immunoregulatory cytokine involved in early Th1 initiation^7^ and can affect T‐cell proliferation and cytotoxic activity.^8^ The influences of IL‐27 on HIV infection and replication have been discussed, an experiment using HIV infected mice as a model proved that IL‐27 could increase dendritic cell functions, in addition, a more direct function of IL‐27 is performed in an in vitro test, which is the inhibition of R5 and X4 HIV replication.^9^

T lymphocyte subsets are the important detection in AIDS testing laboratory. As a significant immune cell in human immune system and the target of HIV, CD4^+^T can directly reflect the immune function of human body, and it is the most definite indicator of immune system damage in patients with HIV infection. Cytotoxic T lymphocytes (CTL), mainly composed of CD8^+^T cells, can actively kill infected cells and suppress viral replication.^10^ Thus, we can get a better understanding of immunological status of body by CD4^+^T and CD8^+^T counts.

In recent years, IL‐27 gene polymorphisms shown in quite a few studies were associated with infectious disease susceptibility.^11^, ^12^, ^13^ Additionally, IL‐27 plasma level becomes lower in HIV‐infected patients, which was proved by Zheng et al.^14^ Moreover, polymorphisms of some genes such as BST2,^15^ BSSL,^16^ APOBEC3H^17^ have been proposed to be related to the progression of HIV infection. We therefore hypothesize that IL‐27 gene polymorphism is associated with HIV infection and its progression. Meanwhile, we detected the serum IL‐27 levels, HIV viral road and counted the absolute CD4^+^T and CD8^+^T cells aiming to investigate the association between IL‐27 gene polymorphism and serum IL‐27 level, HIV viral road and CD4^+^T and CD8^+^T cells in HIV‐infected patients. Based on what were mentioned above, we can get a better understanding of the occurrence and development of the disease about HIV infection, which may provide rationale for clinical diagnosis and treatment.

## 2 MATERIALS AND METHODS

### 2.1 Study subjects

A total of 300 patients with HIV infection whose serum anti‐HIV antibodies were confirmed positive by ELISA and Western blot methods, other infectious or immune diseases excluded, were randomly selected in our study, including 48 long‐term nonprogressors (LTNP) and 252 typical progressors (TP). All patients are untreated before being selected. Moreover, 300 age and gender matched controls were recruited at the same period. The patients and healthy individuals were Chinese and resided in the same geographic area in Guangxi China. This protocol was approved by ethics committee of the Affiliated Hospital of Youjiang Medical University for Nationalities, and written informed consent was obtained from all individuals.

### 2.2 Selection of IL‐27 SNPs

Search IL‐27 gene in NCBI to select the suitable SNPs. Selection criteria are as follows: minor allele frequency (MAF) of IL‐27 SNPs should be >0.05 in Han Chinese data of HapMap database; try to find SNPs in promoter region, exon region, 5′‐UTR, 3′‐UTR; tagging SNPs; refer to the literature on the association of IL‐27 SNPs and other diseases.

### 2.3 DNA extraction and IL‐27 genotyping

Genomic DNA was extracted from peripheral blood by using a whole‐blood genome DNA extraction reagent kit (Yaneng BIOscience [Shenzhen] Co.,Ltd [Shenzhen City, China]). The PCR primers which are presented in Table 1 were designed based on the reference sequence in GenBank and carried out by online primer 3.0 solfware (http://frodo.wi.mit.edu/cgi-bin/primer3/primer3_www.cgi). The genotypes of IL‐27 SNPs (rs17855750, rs181206, rs40837) were detected by multiple single nucleotide primer extension technique and genotyping results were analysed by GeneMapper4.1 (Applied Biosystems). To confirm the genotyping results, PCR‐amplified DNA samples were examined by DNA sequencing method, and the results were 100% concordant.

**Table 1:** The primer sequences used for detecting IL‐27N SNPs

SNP ID	PCR primers
rs17855750	F:5′‐ CTGGGGGGCAAGGTCTGTTAGT‐3′
	R: 5′‐ TCAAGCTGGTGTCTGGGGATTC‐3′
	EF:5′‐ TTTTTTTTTTTTTTTTTTTTTTTCATCTCGCCAGGAAGCTGCTC‐3′
rs181206	F: 5′‐ CAGCTGCATCCTCTCCATGTTG‐3′
	R: 5′‐ GCTGAGGTGGGGGAAGAGCTAC‐3′
	EF: 5′‐ TTTTGGTCCCCAGCCCTCCCAGC‐3′
rs40837	F: 5′‐ GAGGACCAGAGGGGCTTTCAGT‐3′
	R: 5′‐ CCCTGATCGGTGGCTTCTTAGC‐3′
	EF: 5′‐ CCAGCCCCCTGCCCCAGGC‐3′

### 2.4 Serum IL‐27 determination

Blood samples of patients with HIV infection and healthy controls were collected by a serum separator tube, the serum was separated after clot at room temperature, and the freshly prepared serum could be immediately assayed or stored at –80°C until analysis. The serum IL‐27 level was measured by enzyme‐linked immunosorbent assay (ELISA) kit (Biolegend I nc,US) following the manufacturer's protocol. Developed colour reaction was measured at 450 nm on an ELISA reader (RT‐6000, China). The range of detection was 11‐500 pg/mL.

### 2.5 Quantitative determination of HIV viral road

Real‐time PCR was used to detect the HIV viral road in plasma by fluorescence quantitative detection kit (QIAGEN Biological Engineering Co., Ltd., Shenzhen, China) whose linear range is 20‐1 000 000 copies/mL.

### 2.6 CD4^+^T and CD8^+^T count

CD4^+^T and CD8^+^T counts in fresh whole blood were obtained by flow cytometry on BD FACScantoII automatic flow cytometry (Becton Dickinson Immunocytometry System) within eight hours. The reagent is BD Multitest 6‐colour TBNK Reagent produced by Becton, Dickinson and Company, BD Biosciences.

### 2.7 Statistical analysis

Conformity of genotype distribution to Hardy‐Weinbery equilibruim (HWE) was tested by comparing the observed genotype frequencies and expected genotype frequencies by a goodness‐of‐fit *χ* ^2^. Differences in allele frequencies in three SNPs between cases and control were determined using a chi‐square test. Strength of the association between IL‐27 SNPs polymorphism and risk of HIV infection and disease progression was expressed as odds ratio (OR) with 95% confidence interval (95% CI).OR, 95% CI and *P* were adjusted based on gender and age using unconditional logistic regression. Differences between continuous variables (IL‐27 level, HIV viral road, CD4^+^T and CD8^+^T count) were compared using Mann‐Whitney *U* test. Spearman correlation was used to analyse correlation. *P < *0.05 was considered statistical significant. All statistical analyses were done by using SPSS 17.0 statistical software package (SPSS Inc., Chicago, USA).

## 3 RESULTS

### 3.1 Clinical characteristics of the study subjects

The demographics and clinical parameters of HIV‐infected patients and controls in this study are shown in Table 2. There were no significant difference between cases and controls in age (*P = *0.345) and gender (*P = *0.618). Both serum IL‐27 levels and CD4^+^T counts were significantly lower in patients of HIV infection than those in controls (*P *<* *0.001, respectively). Group of HIV infection is divided into group LTNP and TP according to the progression of disease whose demographics and clinical parameters are shown in Table 3. Difference could not be found between LTNPs and TPs in age (*P = *0.450) and gender (*P = *0.149). TPs showed low serum IL‐27 levels, CD4^+^T and CD8^+^T counts compared with LTNPs (*P *<* *0.05, respectively).

**Table 2:** Demographics and clinical parameters of HIV‐infected patients and controls

Variables	HIV‐infected patients (n = 300)	Healthy controls (n = 300)	*P*
Age (y), mean ± SD	48.7 ± 17.0	47.6 ± 12.2	0.345
Gender n (%)			
Male	180 (60.0%)	174 (58.0%)	0.618
Female	120 (40.0%)	126 (42.0%)	
Disease progression n (%)			
LTNP	48 (16.0%)		
TP	252 (84.0%)		
IL‐27 (pg/mL), median	189.25 (75.60‐286.30)	265.80 (229.00‐389.90)	<0.001
HIV viral road (copies/mL), median	510 23.50 (214‐111025)	NA	
CD4^+^T count (cells/mm^3^), median	75.00 (18‐693)	789.50 (457‐2106)	<0.001
CD8^+^T count (cells/mm^3^), median	426.50 (27‐2161)	416.50 (155‐1696)	0.708

NA, not available.

**Table 3:** Demographics and clinical parameters of LTNP and TP

Variable	LTNP group (n = 48)	TP group (n = 252)	*P*
Age (y), mean ± SD	49.0 ± 14.8	47.3 ± 11.6	0.450
Gender n (%)			
Male	30 (62.5%)	150 (59.5%)	0.149
Female	18 (37.5%)	102 (40.5%)	
IL‐27 (pg/mL), median	242.45 (185.00‐286.30)	186.05 (75.60‐254.00)	<0.001
HIV viral road (copies/mL), median	512.50 (214‐2105)	592 55.00 (1230‐111025)	<0.001
CD4^+^T count (cells/mm^3^), median	530.50 (506‐693)	46.50 (18‐390)	<0.001
CD8^+^T count (cells/mm^3^), median	453.00 (327‐1078)	407.00 (27‐2161)	0.038

### 3.2 Association of IL‐27 polymorphism with HIV infection

The genotypes and allele frequencies of IL‐27 rs17855750, rs181206, rs40837 polymorphisms between HIV‐infected patients and controls are shown in Table 4. All genotype distributions in two groups were in Hardy‐Weinbery equilibruim (HWE) (*P *>* *0.05). In rs40837, the AG genotype and AG+GG genotype were associated with susceptibility to HIV infection (AG vs AA: adjusted OR = 1.60, 95% CI, 1.11‐2.30, *P = *0.012; AG+GG vs AA: adjusted OR = 1.44, 95% CI, 1.02‐2.03, *P = *0.038). Nevertheless, no associations were found between rs17855750 and rs181206 polymorphisms and HIV infection (*P *>* *0.05).

**Table 4:** Genotypes and allele frequencies of IL‐27 in HIV‐infected patients and controls

Polymorphism	Cases n = 300 (%)	Controls n = 300 (%)	OR (95% CI)	*P* a
rs17855750				
AA	223 (74.3)	204 (68.0)	1.00 (reference)	
AC	70 (23.3)	90 (30.0)	0.72 (0.50‐1.04)	0.083
CC	7 (2.3)	6 (2.0)	0.98 (0.32‐3.00)	0.970
AC+CC	77 (25.6)	96 (32.0)	0.74 (0.51‐1.06)	0.098
A	516 (86.0)	498 (83.0)	1.00 (reference)	
C	84 (14.0)	102 (17.0)	0.80 (0.58‐1.09)	0.151
rs181206				
AA	165 (55.0)	170 (56.7)	1.00 (reference)	
AG	106 (35.3)	106 (35.3)	1.01 (0.71‐1.43)	0.968
GG	29 (9.7)	24 (8.0)	1.45 (0.81‐2.60)	0.216
AG+GG	135 (45.0)	130 (43.3)	1.09 (0.78‐1.51)	0.624
A	436 (72.7)	446 (74.3)	1.00 (reference)	
G	164 (27.3)	154 (25.7)	1.09 (0.84‐1.41)	0.513
rs40837				
AA	93 (31.0)	117 (39.0)	1.00 (reference)	
AG	162 (54.0)	132 (44.0)	1.60 (1.11‐2.30)	**0.012**
GG	45 (15.0)	51 (17.0)	1.05 (0.64‐1.72)	0.856
AG+GG	207 (69.0)	183 (61.0)	1.44 (1.02‐2.03)	**0.038**
A	348 (58.0)	366 (61.0)	1.00 (reference)	
G	252 (42.0)	234 (39.0)	1.13 (0.90‐1.45)	0.290

Sum of the genotype frequencies may not be 100% due to the rounding at one decimal positions.

OR, odds ratio; 95% CI, 95% confidence interval.

a Adjusted by age and gender.

**Significance of bold values:** Our results have demonstrated that rs40837 polymorphss of IL‐27 were significangtly associated with susceptibility to HIV infection, individuals carrying AG and AG+GG of rs40837 had a increased risk of infecting HIV.

### 3.3 Association of IL‐27 polymorphism with disease progression of HIV infection

The genotypes and allele frequencies of IL‐27 rs17855750, rs181206, rs40837 polymorphisms between LTNPs and TPs are shown in Table 5. All genotype distributions in two groups were in Hardy‐Weinbery equilibruim (HWE) (*P *>* *0.05). In rs40837, the AG genotype was associated with disease progression of HIV infection (LTNP: AG vs AA: adjusted OR = 2.33, 95% CI, 1.13‐4.80, *P = *0.021; TP: AG vs AA: adjusted OR = 1.50, 95% CI, 1.04‐2.24, *P = *0.030). However, no significant associations between rs17855750, rs181206 and disease progression were observed (*P *>* *0.05).

**Table 5:** Genotypes and allele frequencies of IL‐27 in LTNPs and TPs

Polymorphism	Controls n = 300 (%)	LTNP n = 48 (%)	TP n = 252 (%)	LTNP vs Controls OR (95% CI)	*P* a	TP vs Controls OR (95% CI)	*P* a
rs17855750							
AA	204 (68.0)	35 (73.0)	188 (74.6)	1.00 (reference)		1.00 (reference)	
AC	90 (30.0)	10 (20.8)	60 (23.8)	0.70 (0.33‐1.48)	0.343	0.72 (0.49‐1.07)	0.106
CC	6 (2.0)	3 (6.3)	4 (1.6)	2.85 (0.67‐12.11)	0.156	0.62 (0.17‐2.27)	0.470
AC+CC	96 (32.0)	13 (27.1)	64 (25.4)	0.84 (0.42‐1.68)	0.627	1.39 (0.95‐2.04)	0.088
A	498 (83.0)	80 (83.3)	436 (86.5)	1.00 (reference)		1.00 (reference)	
C	102 (17.0)	16 (16.7)	68 (13.5)	0.98 (0.55‐1.74)	0.936	0.76 (0.55‐1.06)	0.108
rs181206							
AA	170 (56.7)	24 (50.0)	138 (54.8)	1.00 (reference)		1.00 (reference)	
AG	106 (35.3)	16 (33.3)	90 (35.7)	1.01 (0.51‐2.00)	0.985	1.00 (0.69‐1.45)	0.998
GG	24 (8.0)	8 (16.7)	24 (9.5)	2.26 (0.90‐5.68)	0.083	1.23 (0.66‐2.30)	0.510
AG+GG	130 (43.3)	24 (50.0)	114 (45.2)	1.23 (0.66‐2.29)	0.508	1.04 (0.74‐1.47)	0.813
A	446 (74.3)	64 (66.7)	366 (72.6)	1.00 (reference)		1.00 (reference)	
G	154 (25.7)	32 (33.3)	138 (27.4)	1.45 (0.91‐2.30)	0.115	1.09 (0.84‐1.43)	0.520
rs40837							
AA	117 (39.0)	12 (25.0)	81 (32.1)	1.00 (reference)		1.00 (reference)	
AG	132 (44.0)	30 (62.5)	132 (52.4)	2.33 (1.13‐4.80)	**0.021**	1.53 (1.04‐2.24)	**0.030**
GG	51 (17.0)	6 (12.5)	39 (15.5)	1.17 (0.41‐3.33)	0.762	1.07 (0.64‐1.79)	0.805
AG+GG	183 (61.0)	36 (75.0)	171 (67.9)	2.00 (1.00‐4.03)	0.051	0.72 (0.50‐1.03)	0.071
A	366 (61.0)	54 (56.3)	294 (58.3)	1.00 (reference)		1.00 (reference)	
G	234 (39.0)	42 (43.8)	210 (41.7)	1.22 (0.79‐1.88)	0.377	1.12 (0.88‐1.42)	0.368

Sum of the genotype frequencies may not be 100% due to the rounding at one decimal positions.

OR, odds ratio; 95% CI, 95% confidence interval.

a Adjusted by age and gender.

**Significance of bold values:** In accordance with our further study, we found that IL‐27 rs40837 polymorphisms may be related to disease progression of HIV infection. When homozygotes of major allele was taken as references, the heterozygote AG of rs40837 showed high OR in the LTNP group and low OR in the TP group. These observations suggest that the majority of patients carrying rs40837 heterozygote stay in the LTNP period compared with TP period.

### 3.4 Association of IL‐27 polymorphism and serum IL‐27 level

**Figure 1:**
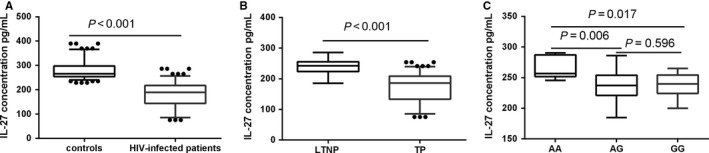
ELISA determination of IL‐27 expression. A, Serum IL‐27 levels in HIV‐infected patients and controls. B, Serum IL‐27 levels in LTNPs and TPs. C, Serum IL‐27 levels in LTNPs with different genotypes of rs40837 (AA, AG, GG). Data were presented as a box plot with 2.5‐97.5 percentile. Statistical tests were performed using Mann‐Whitney *U* test

The median serum IL‐27 level was 189.25 pg/mL (range 75.60‐286.30 pg/mL) in HIV‐infected patients and 265.80 pg/mL (range 229.00‐389.90 pg/mL) in controls. The serum IL‐27 levels in cases were significantly lower than in controls (*P *<* *0.001, Figure 1A). We found no significant associations of three polymorphisms with serum IL‐27 level in cases (*P *> 0.05). When we compared LTNPs to TPs, difference can be observed, the median serum IL‐27 level was 242.45 pg/mL (range 185.00‐286.30 pg/mL) and 186.05 pg/mL (range 75.60‐254.00 pg/mL), respectively (*P* < 0.001, Figure 1B). We further found that LTNPs with rs40837 AG genotype or GG genotype had lower serum IL‐27 levels than with AA genotype (*P *<* *0.05, respectively, Figure 1C), however, there were no difference in the serum IL‐27 level of individuals with AG genotype and GG genotype (*P *>* *0.05). We failed to find any associations of rs17855750 and rs181206 polymorphisms with serum IL‐27 level in LTNPs (*P *> 0.05). There were no correlations between the three polymorphisms and TP (*P *> 0.05).

### 3.5 Association of IL‐27 polymorphisms and HIV viral road

Plasma HIV RNA levels in controls were below detection range. In TPs, there were much higher levels compared to LTNPs, the levels were 59255.00 copies/mL (range 1230‐111 025 copies/mL) and 512.50 copies/mL (range 214‐2105 copies/mL), respectively (*P *<* *0.001). Nevertheless, no association between IL‐27 polymorphisms and HIV viral road was found in the present study (*P *>* *0.05).

### 3.6 Association of IL‐27 polymorphisms and CD4^+^T, CD8^+^T count

**Figure 2:**
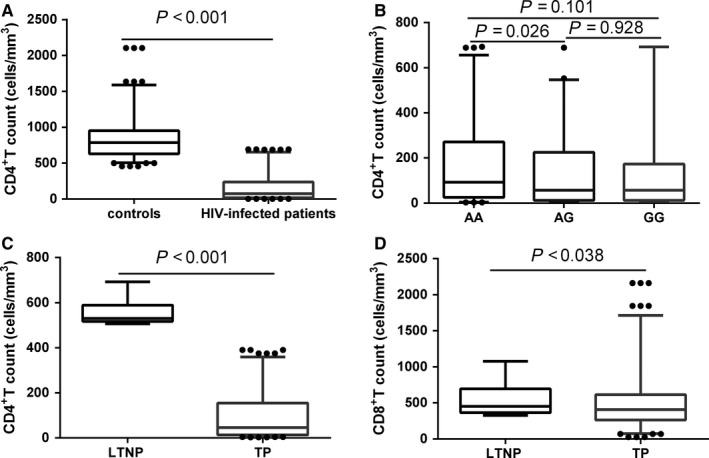
CD4^+^T and CD8^+^T count. A, CD4^+^T counts in HIV‐infected patients and controls. B, CD4^+^T counts in HIV‐infected patients with different genotypes of rs40837 (AA, AG, GG). C, CD4^+^T counts in LTNPs and TPs. D, CD8^+^T counts in LTNPs and TPs. Data were presented as a box plot with 2.5‐97.5 percentile. Statistical tests were performed using Mann‐Whitney *U* test

The median CD4^+^T count was 75.00 cells/mm^3^ (range 18.00‐693.00 cells/mm^3^) in HIV‐infected patients and 789.50 cells/mm^3^ (range 457.00‐2106.00 cells/mm^3^) in controls. The CD4^+^T counts in cases were significantly lower than in controls (*P *<* *0.001, Figure 2A). In contrast, individuals with rs40837 AA genotype had higher CD4^+^T counts than with AG genotype in cases (*P *<* *0.05, Figure 2B). In addition, CD4^+^T counts in TPs were significantly lower than in LTNPs (median was 46.50 vs 530.50 cells/mm^3^, respectively) (*P *<* *0.001, Figure 2C). No significant difference was observed between IL‐27 polymorphisms and CD4^+^T count in LTNP and TP group (*P *>* *0.05). We also analysed association of IL‐27 polymorphisms and CD8^+^T count, no association could be found between them in different group. We only found the difference in CD8^+^T count between LTNP and TP group (*P *<* *0.05, Figure 2D).

### 3.7 Correlations between serum IL‐27 concentration, HIV viral road, CD4^+^T, and CD8^+^T counts

**Figure 3:**
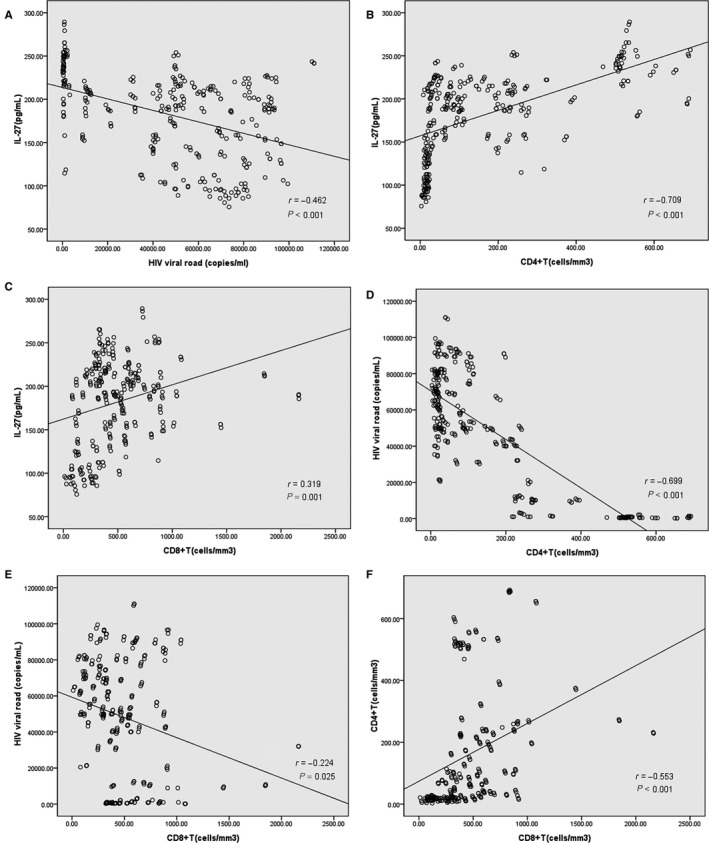
Correlation analysis. A, Serum IL‐27 concentration and HIV viral road. B, Serum IL‐27 concentration and CD4^+^T counts. C, Serum IL‐27 concentration and CD8^+^T counts. D, HIV viral road and CD4^+^T counts. E, HIV viral road and CD8^+^T counts. F, CD4^+^T counts and CD8^+^T counts

Correlation analysis showed that serum IL‐27 concentration was negatively associated with HIV viral road (*r* = –0.424, *P* < 0.001) (Figure 3A), positively associated with CD4^+^T counts (*r* = 0.675, *P* < 0.001) (Figure 3B), and positively associated with CD8^+^T counts (*r* = 0.319, *P* = 0.001) (Figure 3C). The relationships between HIV viral road and CD4^+^T counts and CD8^+^T counts both were negative (*r* = –0.703, –0.226, respectively, *P* < 0.001) (Figure 3D, E). Moreover, CD4^+^T counts was positively associated with CD8^+^T counts (*r* = 0.554, *P* < 0.001) (Figure 3F).

## 4 DISCUSSION

Host genetic variation is an important determinant of HIV infection and disease progression.^18^ Moroever, cytokines are involved early in the pathogenesis of HIV infection and disease progression as a essential component of immunological dysregulation and immunodeficiency and as determinants controlling virus replication.^9^ IL‐27, a cytokine mainly produced by dendritic cells, monocytes, and phagocytic cells, has been shown to interfere HIV‐1 virus replication(against HIV‐1 infection)with controversial findings. In the present study, we explored the role of three variants rs17855750, rs181206, rs40837 of IL‐27 polymorphisms in HIV infection and its progression. To our knowledge, this is the first study reporting the association of IL‐27 gene polymorphisms with susceptibility to HIV infection and disease progression. Our results have demonstrated that rs40837 polymorphsms of IL‐27 were significantly associated with susceptibility to HIV infection, individuals carrying AG and AG+GG of rs40837 had a increased risk of infecting HIV. rs40837 is mapped on 3′‐UTR of IL‐27 gene which is located in the human chromosome 16 and its molecular consequence is 3 prime UTR variant. In other studies, Rosalinda et al^19^ showed that rs40837A alleles were significantly associated with a decreased risk of pCAD, Si F et al^20^ found a significant increase in the risk of Kawasaki disease with coronary arterial lesions in children with rs40837, and Jesus et al^21^ conferred no association between rs40837 and UC, a possible explanation might be rs40837 exists genetic heterogeneity in different diseases. Of note, there are also some researches investigating association between polymorphisms of other interleukins such as IL‐10^22^ and IL‐28B^23^ and HIV infection, and the results are different and even conflicting. In accordance with our further study, we found that IL‐27 rs40837 polymorphisms may be related to disease progression of HIV infection. When homozygotes of major allele was taken as references, the heterozygote AG of rs40837 showed high OR in the LTNP group and low OR in the TP group. These observations suggest that the majority of patients carrying rs40837 heterozygote stay in the LTNP period compared with TP period. In a word, IL‐27 rs40837 is not only associated with susceptibility to HIV, but also determines the progression of disease. Similar to the results found here, it has been suggested that individuals with the homozygous IFN‐874A/A genotype have a significantly higher risk of infection and progression to AIDS.^24^

Interleukin‐27 (IL‐27) can inhibit HIV‐1 replication in activated PBMC, CD4^+^T cells, macrophages, and dendritic cells,^25^, ^26^, ^27^ which may be laterally confirmed by Zheng et al^14^ and our results suggest that IL‐27 concentration was negatively associated with HIV viral road. On the other hand, the serum IL‐27 level showed a down‐regulation in the group of patients in this study as compared with the control group which is consistent with what Zheng et al^14^ discovered, suggesting that IL‐27 might play a protective role in HIV infection. It is reported that genetic polymorphisms of cytokines have been associated with variations in the level of transcription and expression of cytokines that can exert activities in human diseases.^28^Combined with our conclusion that IL‐27 gene polymorphism is associated with HIV infection and its disease progression, we studied the difference in the expression of serum IL‐27 levels in different genotypes of IL‐27, thus exploring the relationship between genetic polymorphism of cytokines, expression of cytokines and diseases. With regard to association between IL‐27 polymorphisms and serum IL‐27 level, no obvious association could be found in cases according to our observation, which was consistent with Rosalinda et al^19^ finding even though the diseases we studied are different. However, serum IL‐27 levels of LTNPs with rs40837 AG genotype or GG genotype were lower than with AA genotype, this finding supported the view that cytokines’ genetic polymorphisms were associated with its expression. Contacted with what is mentioned above IL‐27 rs40837 polymorphisms were associated with LTNP, we assume that cytokine gene mutation affects its expression level, thus guiding the disease progression.

CD4^+^T is the target cell of HIV virus, when the human body is infected with HIV, the primary outcome is that CD4^+^T counts continue to decline, thereby resulting in disease progresses. This was consistent to our finding that HIV viral road was negatively associated with CD4^+^T counts and was confirmed by a previous study.^29^ It is clear that CD4^+^T counts were associated with changes in serum IL‐27,^30^ after positive association between IL‐27 and CD4^+^T counts performed in this study, we were surprised to found the difference of CD4^+^T counts between individuals with homozygotes of major allele AA and heterrozygote AG in IL‐27 rs40837 was obvious, CD4^+^T counts in AG carriers were lower than AA carriers, which might explain the individuals carrying AG of rs40837 had a increased risk of infecting HIV. In a similar literature, Imane Zai et al^23^ have ever investigated the association of IL28B variation and CD4^+^T count in AIDS individuals, and they came to a decision that rs12979860 polymorphism may affect the response to treatment as measured by CD4^+^ T cell counts. On the other hand, evidence have suggested that CD8^+^ T cells are involved in the control of virus replication during HIV infection^31^ and IL‐27 can enhance the activation and proliferation of CTL after being infected by HIV.^32^ Simultaneously, negative relationship between HIV viral road and CD8^+^T counts and positive relationship between IL‐27 concentration and CD8^+^T counts in this study expressed the same meaning with the above two points, which make us believe that the research of relationship between IL‐27 polymorphism and CD8^+^ T count is of significance to the study of HIV infection and its progression. In fact, no correlation has been found in our current studies. Maybe we need repetitive research in future studies. Similar to our study, correlation between IL‐10 polymorphism and CD8^+^ T count was investigated in HIV infection by Felipe Bonfim Freitas et al^22^ and the correlation was proved to be existed between them.

Up to now, only our study has shown that IL‐27 gene polymorphism is associated with HIV infection and disease progression as far as we know. Our data, it is true, showed no direct association between IL‐27 polymorphism and HIV viral road, but we suspect that IL‐27 rs40837 polymorphism might influence the susceptibility to HIV infection and disease progression probably by regulating the level of serum IL‐27 or the quantity of CD4^+^T. However, the specific mechanism is not yet clear and more researches, especially for different countries, different races are warranted to verify our view as a result of the genetic heterogeneity in different ethnic groups. Finally, it should be noted here that, although the results of our study could not completely elucidate the mechanism of HIV infection and disease progression, it can provide a useful direction for further research mechanism.

## CONFLICT OF INTEREST

The authors declare no competing financial interests.
